# Profiling genome-wide DNA methylation

**DOI:** 10.1186/s13072-016-0075-3

**Published:** 2016-06-29

**Authors:** Wai-Shin Yong, Fei-Man Hsu, Pao-Yang Chen

**Affiliations:** Institute of Plant and Microbial Biology, Academia Sinica, Taipei, 11529 Taiwan, ROC; Graduate School of Frontier Sciences, The University of Tokyo, Chiba, 277-8561 Japan

**Keywords:** DNA methylation, Bisulfite sequencing, Hydroxymethylation, Single-cell, Methylome, WGBS, RRBS

## Abstract

DNA methylation is an epigenetic modification that plays an important role in regulating gene expression and therefore a broad range of biological processes and diseases. DNA methylation is tissue-specific, dynamic, sequence-context-dependent and trans-generationally heritable, and these complex patterns of methylation highlight the significance of profiling DNA methylation to answer biological questions. In this review, we surveyed major methylation assays, along with comparisons and biological examples, to provide an overview of DNA methylation profiling techniques. The advances in microarray and sequencing technologies make genome-wide profiling possible at a single-nucleotide or even a single-cell resolution. These profiling approaches vary in many aspects, such as DNA input, resolution, genomic region coverage, and bioinformatics analysis, and selecting a feasible method requires knowledge of these methods. We first introduce the biological background of DNA methylation and its pattern in plants, animals and fungi. We present an overview of major experimental approaches to profiling genome-wide DNA methylation and hydroxymethylation and then extend to the single-cell methylome. To evaluate these methods, we outline their strengths and weaknesses and perform comparisons across the different platforms. Due to the increasing need to compute high-throughput epigenomic data, we interrogate the computational pipeline for bisulfite sequencing data and also discuss the concept of identifying differentially methylated regions (DMRs). This review summarizes the experimental and computational concepts for profiling genome-wide DNA methylation, followed by biological examples. Overall, this review provides researchers useful guidance for the selection of a profiling method suited to specific research questions.

## Background

DNA methylation, one of the most studied epigenetic modifications, involves the addition of a methyl group to the fifth carbon of cytosine (C), forming 5-methylcytosine (5mC), catalyzed by DNA methyltransferases (Dnmts) [1]. DNA methylation predominantly occurs in CpG dinucleotides (CpGs) but is also found less frequently in non-CpG contexts (e.g., CHG and CHH, where H = A, T or C). These contexts affect gene function and structure differently [2]. The de novo DNA methyltransferases Dnmt3a and Dnmt3b are responsible for catalyzing the methylation of Cs, and the maintenance methyltransferase Dnmt1 enables the propagation of DNA methylation patterns during cell division [3–5]. DNA methylation has been associated with numerous cellular processes, such as transcriptional repression, X chromosome inactivation, embryonic development, genomic imprinting, the alteration of chromatin structure and transposon inactivation [6, 7]. The methyl marks are heritable, that certain methylation patterns have transgenerational effects [8]. The patterns of these marks are also dynamically remodeled during distinct reprogramming phases throughout the life cycle of an organism [9]. DNA methylation does not occur exclusively on C residues; methylation can also present as N^6^-methyladenine (6mA) in *Chlamydomonas reinhardtii* (algae) [10], *Caenorhabditis elegans* (nematode) [11], *Drosophila melanogaster* (insect) [12] and vertebrates such as *Xenopus laevis*, mouse and human [13]. In addition to 5mC, 5-hydroxymethylcytosine (5hmC) is another epigenetic mark in the mammalian genome associated with DNA demethylation. 5hmC is produced via the oxidation of 5mC catalyzed by the ten-eleven translocation (TET) family of proteins, and subsequent oxidation results in the formation of 5-formylcytosine (5fC) and 5-carboxylcytosine (5caC) [14].

DNA methylation patterns vary across organisms. The mammalian genome is usually highly methylated; in human embryonic stem cells (hESCs), DNA methylation occurs in up to 80 % of CpGs, with the remaining unmethylated CpG residues enriched in CpG islands (CGI) located at gene promoters [15]. Extremely low methylation levels have been reported in invertebrates such as *Drosophila* [16] and *Bombyx* [17]. In plants, the methylation level varies in the CpG and non-CpG sites; the levels were found to be 24 % CpG, 7 % CHG and 2 % CHH in Arabidopsis [18] and 86 % CpG, 74 % CHG and 5 % CHH in unfertilized ears of maize [19]. Non-CpG methylation plays key roles in plants, in which this modification can silence exogenous DNA via an RNA-dependent DNA methylation pathway (RdDM) [20]. In fungi, the methylation in black truffle is found exclusively in transposons and is absent from genes [21].

Promoter methylation can potentially down-regulate gene expression by altering the chromatin structure and blocking transcription initiation [7]. For example, in mammals, most CGIs in promoters are unmethylated to facilitate binding between proteins and promoter DNA. Positive correlations between active transcription and gene body methylation have been observed in the active X chromosome [22, 23]. Gene body methylation may also function to silence repetitive DNA elements found within the gene body [24]. In addition, gene body methylation has been found to exhibit dramatic changes at intron–exon boundaries, suggesting an association with splicing [25]. Maunakea et al. found that DNA methylation modulates alternative splicing by recruiting methyl-CpG-binding protein MeCP2 to promote exon recognition [26]. In maize, CpG methylation in transcribed regions is positively correlated with transcription, whereas CHG methylation is negatively correlated [27]. Methylation changes at the intron–exon boundaries have also been observed, suggesting that maize DNA methylation is likely associated with alternative splicing [28].

**Fig. 1:**
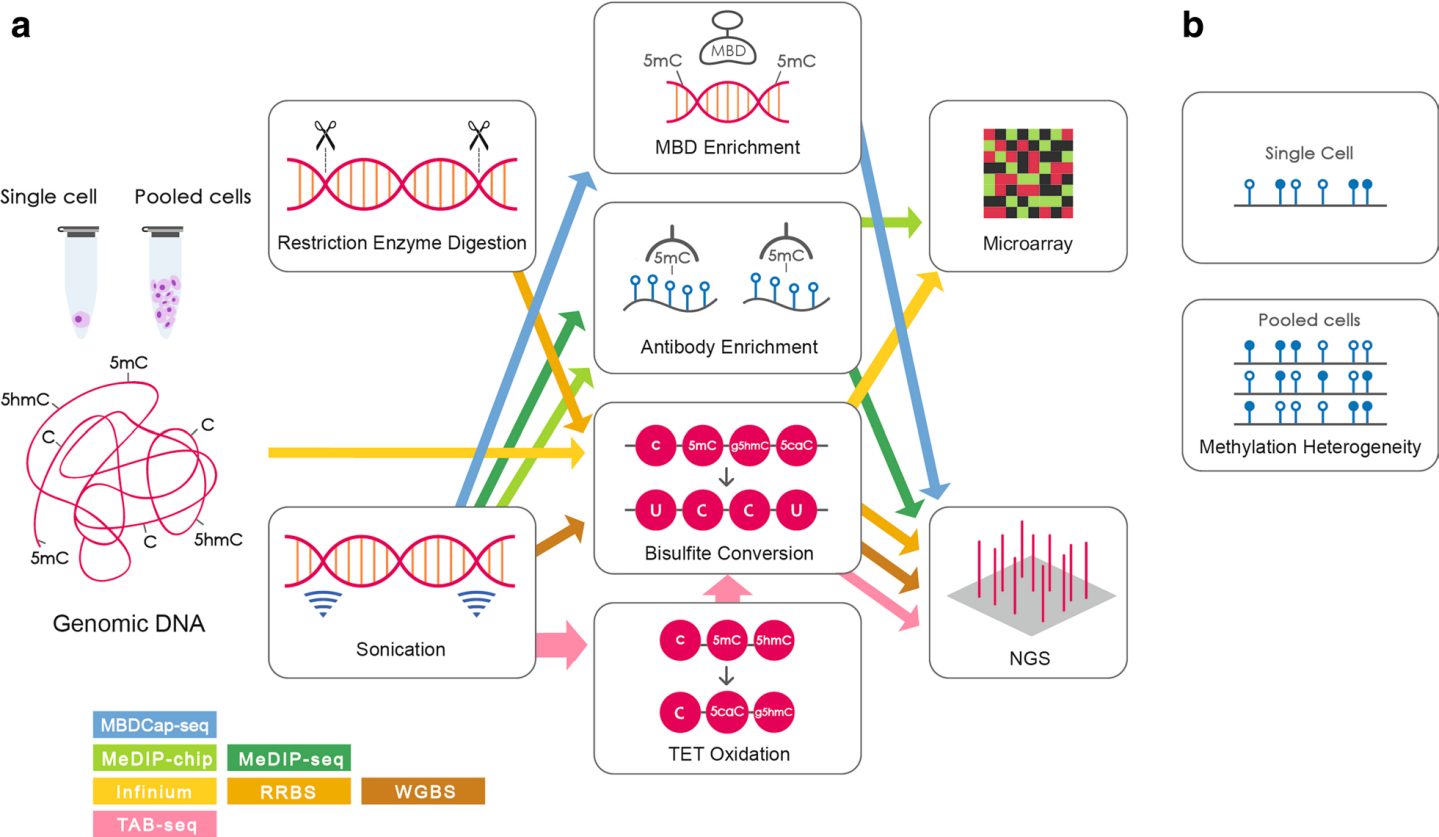
Commonly used methods for genome-wide DNA methylation analysis. **a** The procedures may involve fragmentation of genomic DNA by restriction enzyme digestion or sonication. The genomic DNA can be subjected to MBD enrichment, antibody enrichment, bisulfite conversion or TET oxidation before analyzing by microarray or next-generation sequencing platform. **b** Single-cell DNA methylation analysis that involves the isolation of single cells allows the assessment of methylation heterogeneity in cell populations while other genome-wide DNA methylation profiling methods using pooled heterogeneous cell populations are not capable to dissect the methylation heterogeneity. *Blue concrete dots* represent 5mC, and *hollowed* ones represent C. Each track represents 1 read

These important findings regarding DNA methylation would not have been possible without the advancement of various profiling approaches, both experimental and computational. The accelerated development of array and sequencing technologies has significantly improved DNA methylation profiling, providing an unprecedentedly comprehensive view of the DNA methylation landscape. This review provides an overview of the major profiling approaches, with a focus on the recent and promising genome-wide methodologies (see Fig. 1 for a schematic of the major profiling methods).

## Experimental techniques for DNA methylation profiling

**Fig. 2:**
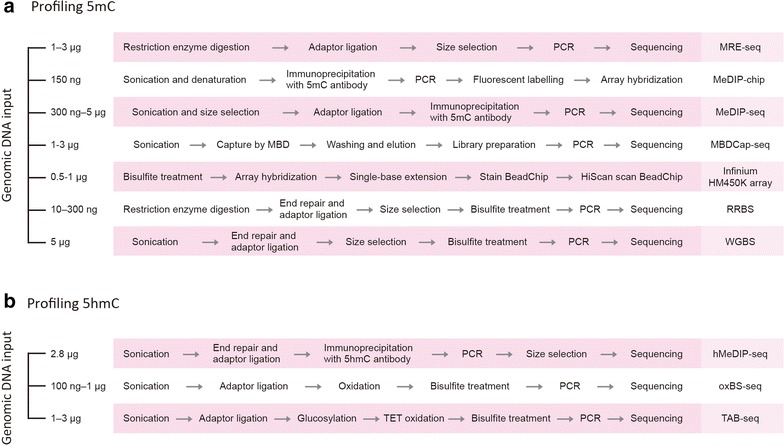
Schematic overview of genome-wide DNA methylation profiling methods. **a** 5mC assays. **b** 5hmC assays. The actual sample requirement may vary according to the type of sample, genome size and number of PCR cycles

Early studies of DNA methylation focused on determining the methylation status of the genes of interest and quantifying the total amount of 5mC [29]. Due to the use of microarray hybridization technology, the study of DNA methylation was able to scale up to the genome-wide level. Next-generation sequencing platforms now allow the construction of genomic maps of DNA methylation at single-base resolution [30]. In the following review, we categorize these experimental approaches into *enzyme digestion, affinity enrichment* and *bisulfite conversion* and introduce the major methods with their advantages and disadvantages (see Table 1). We also include corresponding biological examples for each method in Table 1 to help readers select suitable profiling methods. Figure 2 shows the workflow of the experimental pipelines with the DNA input requirements. Finally, we introduce the most recent development in the epigenomic profiling of a single-cell methylome, 5hmC and the use of third-generation sequencing in detecting DNA methylation in real time.

**Table 1:** Experimental approaches for profiling genome-wide DNA methylation

Experimental approach	Strength	Weakness	Resolution	Quantitative nature	Cost	Examples	References
CHARM	-Cost-effective -Interrogate CpG sites genome-wide irrespective of proximity to genes or CpG islands	-Moderate resolution -Limited to regions in proximity to enzymes’ recognition sites	–	Abundance	Low	CGI shores show alteration DNA methylation in colon cancer [36]	[34]
MBDCap-Seq	-Cost-effective -Allow the detection of DMRs within highly CpG-dense regions and regions with lower CpG density -MBD proteins can discriminate 5mC from 5hmC -No mutation introduced -More sensitive than MeDIP in regions with higher CpG density	-Relatively low resolution -Biased toward hypermethylated regions	~150 bp	Abundance	Moderate	Confirmed previous known differentially methylated sites and discovered new differentially methylated loci in 3 isogenic colon cancer cell lines [38]	[37]
MeDIP	-Cost-effective -No mutation introduced -Specific to 5mC/5hmC depending on the antibody specificity -More sensitive in regions with low CpG density than MBDCap-Seq	-Biased toward hypermethylated regions -Do not identify individual 5mC sites -Inability to predict absolute methylation level	~100 bp	Abundance	Moderate	MBDCap-seq shows higher genomic coverage than MeDIP-seq along with twice as many DMRs between colon cancer and adjacent normal cells [45]	[39, 42]
Illumina’s Infinium Methylation assay	-Cost-effective -Do not require a large amount of input DNA	-Human sample only -Coverage is highly dependent on the array design -Substantial DNA degradation after bisulfite treatment	Single base	Abundance	Low	DNA methylation as a signature to surrogate different cord blood cell types [49]	[47]
WGBS	Evaluate methylation state of almost every CpG sites	-High cost -Substantial DNA degradation after bisulfite treatment -Cannot discriminate between 5mC and 5hmC	Single base	Digital	High	Bulk methylation level of CpG/CHG/CHH of wild-type Arabidopsis and methyltransferase-deficient mutants [18] Genome-wide methylation pattern and site-specific methylation [18] Global demethylation in the endosperm compared to the embryo [55]	[52]
RRBS	-High CGI coverage -High sensitivity -Cost-effective comparing to WGBS	-May exhibit a lack of coverage at intergenic and distal regulatory elements -Substantial DNA degradation after bisulfite treatment -Limited to regions in proximity to enzymes’ recognition sites -Cannot discriminate between 5mC and 5hmC	Single base	Digital	Moderate	The EWAS study integrating DNA methylation, gene expression, proteomics, metabolomics and clinical traits in 90 mouse inbred strains [62]	[60, 61]
scWGBS	Able to study methylome intra-population distribution	-Low sequencing efficiency (~20 million reads typically required per cell) -Cannot discriminate between 5mC and 5hmC	Single base	Digital	High	Determining epigenomic cell-state dynamics in mouse pluripotent and differentiating cells [74]	[72, 74]
scRRBS	-Highly sensitive -Can detect target CpG sites at high coverage with relatively low number of sequence reads	-Substantial DNA degradation after bisulfite treatment -Cannot discriminate between 5mC and 5hmC -Provide relatively poor coverage for imprinting loci	Single base	Digital	High	Profiling epigenomic dynamics of 1 million CpG sites during early embryonic development in ESCs [70]	[70]
TAB-seq	Can distinguish 5hmC from 5mC	-Substantial DNA degradation after bisulfite treatment -Tet enzyme with low efficiency might leave methylated residues unconverted -High sequencing depth is required to detect 5hmC with low abundance	Single base	Digital	High	Profiling 5hmC distribution in 108 days human PGCs to reveal DNA demethylation [83]	[81]

### Restriction enzyme-based methods

Restriction enzyme-based methods take advantage of the differential digestion properties of isoschizomers and neoschizomers. A pair of isoschizomers recognizes the same sequence and has the same point of cleavage but exhibit different sensitivities to the DNA methylation state. Methylation-sensitive restriction enzymes (MREs), such as *Bst*UI, *Hpa*II, *Not*I and *Sma*I, cleave only their unmethylated target sequences (see [31] for lists of MREs) and leave the methylated DNA intact. MRE digestion has been coupled with sequencing technologies to predict genome-wide DNA methylation levels [32]. In the workflow of MRE digestion followed by sequencing (MRE-seq), the MRE cleaves the unmethylated CpG sites of genomic DNA, and the resulting DNA fragments are size-selected and sequenced. The sequencing results reveal the locations of the unmethylated CpG sites within the recognition sites of the enzyme utilized [33]. MRE-seq allows the estimation of relative DNA methylation levels but has relatively low coverage of the genome because the CpG-containing recognition sites are limited.

#### Comprehensive high-throughput arrays for relative methylation (CHARM)

The comprehensive high-throughput arrays for relative methylation (CHARM) method first uses McrBC, an enzyme that digests methylated DNA, to fractionate DNA and subsequently utilizes array hybridization [34]. McrBC recognizes R^m^C(N)_55–103_R^m^C and cleaves half of the methylated DNA and all the methylated CGIs [35], and thus, relatively unmethylated DNA will be size-selected and hybridized to the array. Using CHARM, Irizarry et al. discovered that most DNA methylation differences between colon cancer and adjacent normal tissues occurred in sequences up to 2 kb away from CGIs, termed CpG island shores (CGI shores) [36]. Unexpectedly, differentially methylated regions (DMRs) in CGI shores have a strong inverse relationship with differential gene expression. CHARM, as a restriction enzyme-based method, is able to detect DMRs at CGI shores, which are otherwise not detectable with CpG-directed enrichment methods such as methylated DNA immunoprecipitation (MeDIP).

### Affinity enrichment-based methods

Affinity enrichment-based methods use either methyl-CpG-binding domain (MBD) proteins or antibodies specific for 5mC (as in MeDIP) to enrich methylated DNA regions. The results from an MBD protein-based approach, which relies on the capacity of MBD proteins to bind specifically to methylated DNA sequences, could be profiled using microarray (MBD-chip) or sequencing (MBDCap-seq/MethylCap-seq [37], methylated DNA capture by affinity purification) technologies. Serre et al. used MBDCap-seq to study 3 isogenic colon cancer cell lines, and the results confirmed known methylated loci and regions and identified differential methylation in *ZEB1*, *VASH2* and *PODXL2* between the HCT116 and DICER1-truncated DICER^ex5^ cell lines [38].

#### Methylated DNA immunoprecipitation (MeDIP)

MeDIP utilizes an anti-methylcytosine antibody to immunoprecipitate DNA with methylated CpG sites [39]. The DNA fractions enriched by MeDIP can be evaluated using tiling arrays (MeDIP-chip) or high-throughput sequencing (MeDIP-seq) [40]. MeDIP-seq typically yields a resolution of 100–300 bp and could not discriminate methylation context. This can be an issue when research topics are context-specific. Because the methylation statuses of neighboring CpG sites are correlated, MeDIP-seq can be a cost-effective approach when single-base resolution is not desired [41] (Table 1). Taiwo et al. reported that a minimum of 1× coverage can cover up to 70 % of all CpGs in human, suggesting that the majority of the methylated CpGs can be interrogated by MeDIP given that 60–80 % of the CpGs are methylated in a genome [42]. MeDIP-seq generates the relative enrichment of methylated DNA across the genome, instead of predicting the absolute DNA methylation level. MeDIP-seq is feasible with even a low amount of starting DNA material (as low as 1 ng); therefore, this method can be utilized to profile DNA methylation in minute DNA samples, rare cell types and microdissected tissues [39, 42].

The affinity enrichment-based methods tend to exhibit biases associated with CpG density and copy number variation. For example, in MeDIP CpG-rich fragments are more likely to be enriched than CpG-poor ones, even when they are both fully methylated [43]. Hence, a computational correction such as BATMAN tool, which attempts to normalize CpG content across a wide range of CpG densities, is needed [44]. Moreover, CpG density might directly cause PCR bias due to the strong hydrogen bond between the CG pair [43]. Bock et al. benchmarked MeDIP-seq and MBDCap-seq [45] and found that MethylCap-seq covers more genomic regions than MeDIP-seq, and MBDCap-seq could detect nearly twice as many DMRs as MeDIP-seq at comparable sequencing depth. In CpG-poor regions, both MeDIP-seq and MBDCap-seq show low statistical power to detect DMRs.

### Bisulfite conversion-based methods

Treating genomic DNA with sodium bisulfite deaminates unmethylated C to uracil (U), while methylated C residues remain unaffected [46]. The U eventually converts to thymine (T) in a subsequent polymerase chain reaction (PCR). Bisulfite conversion-based methods provide single-base resolution and are commonly used to investigate specific DNA sequences when coupled with Sanger sequencing. This type of method is also used to study genome-wide methylation via a methylation array, whole-genome bisulfite sequencing (WGBS) and reduced-representation bisulfite sequencing (RRBS) (see Table 1; Figs. 1, 2).

#### Methylation array

Illumina’s Infinium HumanMethylation450 BeadChip (HM450K) protocol involves the bisulfite conversion of genomic DNA and amplification, followed by the hybridization of the converted DNA to arrays containing predesigned probes to distinguish between methylated and unmethylated Cs (Fig. 2). Each HM450 K BeadChip can interrogate more than 450,000 methylation sites that cover 96 % of the CGIs, 92 % of the CGI shores and 86 % of the CGI shelves (2–4 kb from a CGI) [47]. To date, HM450 K arrays dominate studies investigating the cancer methylome [48] and other epigenome-wide studies. For example, Bakulski et al. isolated 7 cord blood cell types, which were compared according to their specific methylation signatures; these authors found that nucleated red blood cells had the most pronounced differences [49]. The most recent implementation of the Infinium^®^ technology, Infinium MethylationEPIC BeadChip, covers more than 850 K CpG methylation sites, including >90 % of the 450 K sites plus additional CpG sites in the enhancer regions identified by the ENCODE and FANTOM5 projects [50].

#### Whole-genome bisulfite sequencing (WGBS)

WGBS (BS-seq; MethylC-seq) theoretically covers all the C information [51]. In this method, genomic DNA is purified and sheared into fragments. The fragmented DNAs are end-repaired; adenine bases are added to the 3′ end (A-tailing) of the DNA fragments, and methylated adapters are ligated to the DNA fragments [52]. The DNA fragments are size-selected before sodium bisulfite treatment and PCR amplification, and the resulting library is sequenced. It should be noted that a high number of PCR cycles and inappropriate selection of a uracil-insensitive DNA polymerase may result in an over-representation in the methylated DNA data [53]. Starting with sufficient genomic DNA may avoid a loss of information from regions of interest and over-amplification. The major advantage of WGBS is its ability to assess the methylation state of nearly every CpG site, including low-CpG-density regions, such as intergenic ‘gene deserts’, partially methylated domains and distal regulatory elements. It can also determine absolute DNA methylation level and reveal methylation sequence context. The first WGBS in 2008 reported the composition of CpG, CHG and CHH methylation in the Arabidopsis genome, the bulk methylation level within each context, and the global methylation pattern in wild-type and methylation-related mutants, as well as specific sites associated with gene expression [18, 54]. In 2013, two maize studies reported that the maize genome is highly methylated, and a specific ‘CHH island’ was found upstream of transcription start sites (TSSs) [19, 28]. In addition to global pattern identification, users could determine regions or even loci with differential methylation between groups using bioinformatics tools. For example, Hsieh et al. compared Arabidopsis endosperm and embryo methylomes and found that virtually the entire endosperm genome is demethylated, coupled with extensive local non-CpG hypermethylation of small interfering RNA-targeted sequences [55]. Lu et al. performed WGBS of maize embryo and endosperm, and the results revealed hypomethylation in the endosperm compared to the embryo [27].

WGBS has become the standard profiling method in major epigenome consortiums, such as NIH Roadmap [56], ENCODE [57], Blueprint [58] and IHEC [59]. For studies interested in regions outside of CGIs, targeted approaches such as reduced-representation bisulfite sequencing (RRBS), MeDIP and MethylCap are not applicable, and the best choice is likely to be WGBS.

#### Reduced-representation bisulfite sequencing (RRBS)

To investigate the mammalian methylome at a lower cost, Meissner et al. developed RRBS, which integrates *Msp*1 restriction enzyme digestion, bisulfite conversion and next-generation sequencing for the analysis of methylation patterns of specific fragments [60]. A size selection of *Msp*I-digested fragments between 40 and 220 bps was found to cover 85 % of CGIs, mostly in promoters, which compose only 1–3 % of the mammalian genome, thereby significantly decreasing the amount of sequencing [51, 61]. RRBS-based protocols are more cost-effective than WGBS because these methods focus on the enrichment of CpG-rich regions in close proximity to the restriction enzyme’s recognition sequence; however, these protocols may exhibit a lack of coverage at intergenic and distal regulatory elements that are relatively less studied.

RRBS has been widely used in profiling large-scale samples. Orozco et al. performed RRBS in 90 inbred mouse strains, conducted an integrative analysis that included genome-wide expression levels, proteomics, metabolomics, and 68 clinical traits, and performed epigenome-wide association studies (EWAS) [62]. They found associations with numerous clinical traits, including bone density, insulin resistance, expression, and protein and metabolite levels. RRBS has also been used in non-mammalians, such as zebrafish [63], wasp [64], oak populations [65] and *Brassica rapa* [66].

#### Commercial DNA methylation assay kits

Another concern for BS-seq is that a large amount of high-quality genomic DNA, e.g., usually 5 μg, is required for WGBS, and RRBS requires 0.01–0.3 μg [51] (see Fig. 2). To study samples with a preciously small amount of DNA, e.g., primordial germ cells (PGCs) and cancer cells, commercial kits for ultralow input were developed. The Ovation^®^ Ultralow Methyl-Seq Library System requires only 10 ng of DNA to construct the WGBS library [67], and the Ovation^®^ RRBS Methyl-Seq Library System requires 100 ng of DNA for RRBS.

For targeted bisulfite sequencing, the SeqCap Epi System from Roche enables the enrichment of a small fraction of the genome containing regions of interest after bisulfite conversion [68]. In addition, the SeqCap Epi CpGiant Enrichment Kit allows the interrogation of more than 5.5 million CpGs in the human genome with a starting DNA input of 1 µg. Roche also provides customization of probe pools according to the type of organism and regions of interest. The SureSelect^XT^ Methyl-Seq Target Enrichment Kit from Agilent Technologies involves the hybridization and enrichment of sequencing libraries with oligonucleotide baits before bisulfite conversion [69]. This platform supports the enrichment of an 84-Mb target covering 3.7 million CpG sites with a DNA input as low as 1 µg.

#### Single-cell methylome

Most genome-wide DNA methylation profiling techniques have common limitations: the need for bulk cell populations as starting materials and the inability to assess methylation heterogeneity among individual cells [70, 71]. To address these issues, single-cell bisulfite-based techniques have been developed. First, single-cell reduced-representation bisulfite sequencing (scRRBS) integrates the steps of *Msp*I digestion to bisulfite conversion into one tube of cell lysate to minimize DNA loss and to provide methylation information on approximately 1 million CpG sites within an individual mouse or human cell [70]. Another single-cell DNA methylation analysis method, namely single-cell bisulfite sequencing (scBS-seq), is a modified post-bisulfite adapter tagging (PBAT) protocol [72, 73]. PBAT circumvents the issue of a massive bisulfite-induced loss of sequencing templates in WGBS by performing bisulfite treatment ahead of adapter tagging, thereby enabling the use of a lower starting amount of DNA (only 100 ng) and eliminating the need for global amplification [73]. scBS-seq enables the measurement of DNA methylation at up to 48.4 % of the CpG sites and was reported to achieve higher recovery rates than scRRBS [71, 72]. Farlik et al. described single-cell whole-genome bisulfite sequencing (scWGBS) of human and mouse cells and bioinformatics inferences for epigenomic cell-state dynamics in pluripotent and differentiating cells [74]. These single-cell techniques can be applied in studies involving limited cell amounts and heterogeneous cell populations [71, 72] and are particularly useful for specific cell types that play important roles in early development, such as sperm cells, oocytes, PGCs and embryonic stem cells (ESCs).

### Genome-wide 5-hydroxylmethylation profiling

The TET family of dioxygenases catalyze the oxidation of 5mC to 5hmC. The detection of 5hmC gained much attention recently after this C modification was identified as an epigenetic mark in mammals (mouse brain and ESCs), and 5hmC has been reported to be an intermediate in DNA demethylation [75, 76]. The detection of 5hmC is technically more challenging than that of 5mC due to the low abundance of 5hmC, and standard bisulfite sequencing does not distinguish between 5mC and 5hmC because both are resistant to bisulfite treatment [77]. Hydroxymethylated DNA immunoprecipitation (hMeDIP), which is modified from MeDIP, characterizes the relative abundance of 5hmC at specific loci or throughout the entire mammalian genome. hMeDIP involves immunoselection and immunoprecipitation using anti-5hmC antibodies and subsequent analysis by qPCR, microarray hybridization or next-generation sequencing [78].

#### Oxidative bisulfite sequencing (OxBS-seq)

A modified bisulfite sequencing technique, oxidative bisulfite sequencing (OxBS-seq) distinguishes between 5mC and 5hmC via the highly selective chemical oxidation of 5hmC to 5fC [79]. After bisulfite treatment, 5fC is converted to U and is read as T in the sequencing stage. Unlike 5hmC, 5mC does not undergo oxidation upon bisulfite treatment and will be detected as C after sequencing. The 5hmC level can be quantified by comparing the data from BS-seq (which identifies both 5hmC and 5mC) and the data from OxBS-seq (which identifies 5mC). The disadvantages of this technique are the oxidative degradation of DNA and the requirement for multiple bisulfite treatments to completely deaminate 5fC [80].

#### TET-assisted bisulfite sequencing (TAB-seq)

TET-assisted bisulfite sequencing (TAB-seq) has been used to generate genome-wide 5hmC profiles at a single-base resolution in human and mouse ESCs [81]. In TAB-seq, 5hmC is protected from TET protein-mediated oxidation by the addition of glucose to 5hmC using β-glucosyltransferase (β-GT) to generate β-glucosyl-5-hydroxymethylcytosine (g5hmC). 5mC is oxidized by the Tet1 enzyme to 5caC. 5caC and unmethylated C are susceptible to bisulfite conversion and thus are sequenced as T, whereas 5hmC is sequenced as C. TAB-seq measures 5hmC directly, and information regarding 5mC can be obtained using the same analysis pipeline as BS-seq. Highly active TET proteins are required for the efficient conversion of 5mC to 5caC (more than 96 %), or else the incomplete conversion of 5mC might lead to false identification as 5hmC sites [81]. Both oxidative bisulfite conversion and TET-assisted bisulfite conversion are compatible with microarray and sequencing platforms to generate the 5hmC methylation profile for a whole genome or targeted regions [82]. The relatively low levels of 5hmC and the subtraction step demand an increase in the sequencing coverage and the number of replicates. A study of human PGC epigenome used TAB-seq to reveal the demethylation  during epigenetic reprogramming between 57 and 113 days, and the heterogeneity of 5hmc in both individual loci and at individual cells has been identified [83].

### Third-generation sequencing

Emerging third-generation sequencing technologies [84], including single-molecule real-time sequencing (SMRT) and Oxford Nanopore technology, have been recently adopted in epigenetics research.

#### Single-molecule real-time sequencing

Developed by Pacific Biosystems, SMRT allows the direct detection of base modifications by monitoring the activity of DNA polymerase during the incorporation of different fluorescently labeled nucleotides into complementary DNA strands [85, 86]. The direct detection of various base modifications involves the measurement of the kinetics variation in the time between base incorporations. This technology has the following advantages over second-generation sequencing: (1) minimal chemical modification during library preparation; (2) the requirement for DNA amplification is eliminated; (3) reduced requirement for input DNA; (4) the ability to generate longer reads (average read length of 3000 bp); and (5) the ability to detect different types of epigenetic modifications [86, 87]. SMRT has been used in the identification of 6mA in C. *elegans*, and the recently developed SMRT of chromatin immunoprecipitation enriched DNA (SMRT-ChIP) has resulted in the identification of 6mA and associated demethylase *ALKBH1* in mouse ESCs [11, 88].

#### Nanopore sequencing

In nanopore sequencing, single-stranded DNA is pulled by a phage DNA polymerase through a bacterial pore in single-nucleotide steps, and the ion current through the pore is recorded [89]. C can be distinguished from 5mC and 5hmC based on differences in the current traces. Although the detection of 5mC and 5hmC using nanopore sequencing yielded encouraging results for the DNA methylation profiling of a single locus, the application of this method to genome-wide DNA methylation profiling has yet to be established.

Taken together, these applications of third-generation sequencing open doors to more discoveries of different epigenetic modifications and potentially reveal the novel functions of these epigenetic marks in gene expression. Despite its many promising features, the broad application of third-generation sequencing is still limited by a higher error rate, higher cost and lower throughput than second-generation sequencing technologies [90]. The throughput and accuracy must be substantially improved before applying these approaches to studies involving complex genomes.

## Bioinformatics analysis of WGBS and RRBS

**Fig. 3:**
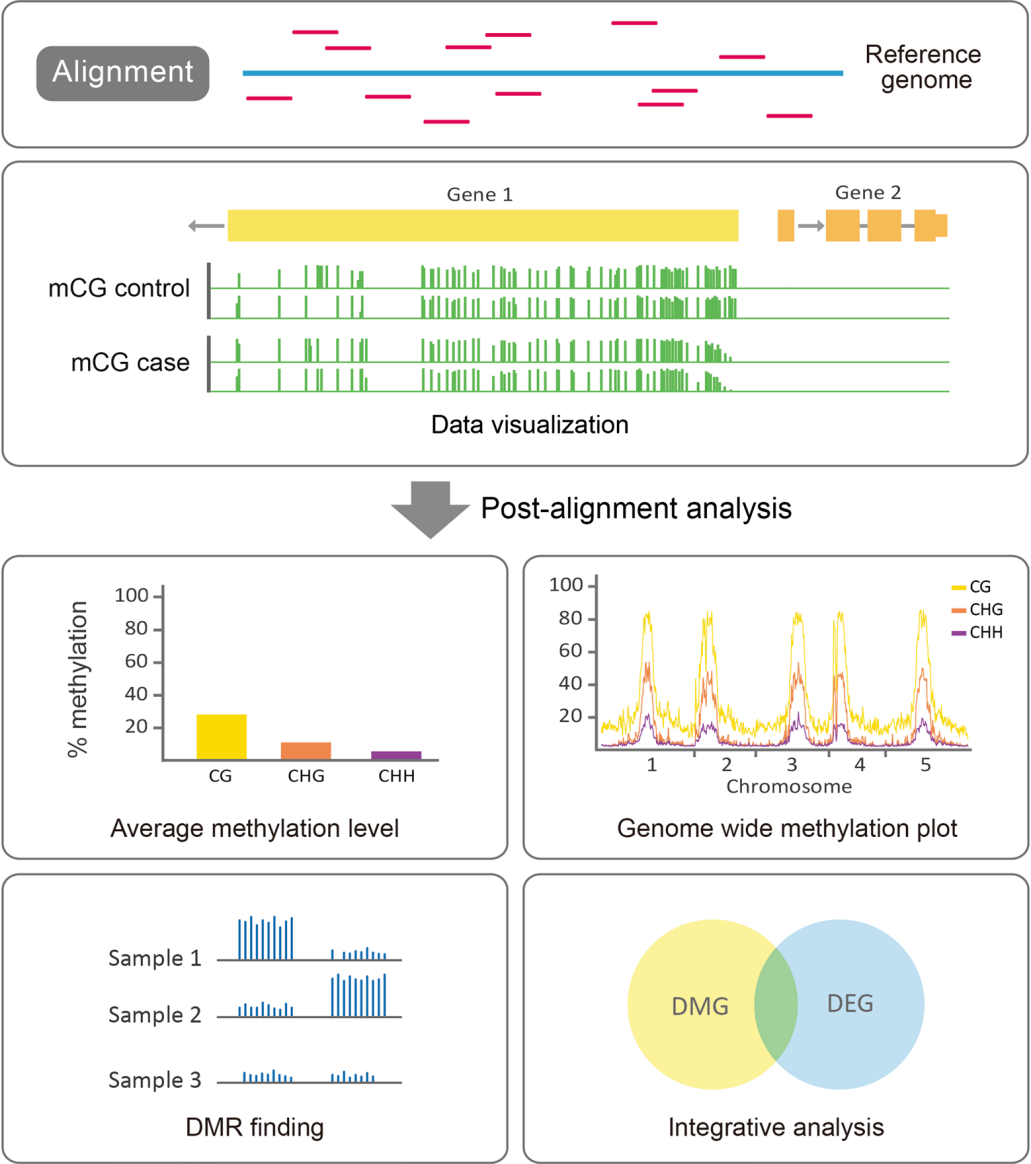
Computational pipeline for genome-wide bisulfite sequencing data analysis. Reads from bisulfite sequencing are first aligned to the reference genome. The alignment data may be visualized in different tracks for comparison. After methylation calling, the bulk methylation level and genome-wide methylation level are calculated and plotted, and DMRs are determined. To perform an integrative analysis, DNA methylation data are coupled with gene expression, e.g., differentially expressed genes (DEGs), to delineate the regulatory role of DNA methylation

The general workflow for the bioinformatics analysis of DNA methylation data includes data processing, the quantification of DNA methylation levels, general profiling, the identification of DMRs and the visualization of the methylome [91]. Array-based data, such as that from Illumina’s HM450K, are fluorescence intensities that quantify the relative abundance of methylated and unmethylated loci. The data from other non-bisulfite-conversion methods, such as MRE-seq and MeDIP-seq, are usually analyzed by comparing the relative abundance of fragments. Bisulfite-converted data, such as those from WGBS and RRBS, involve methylation calling at individual Cs, and statistical testing is required to assess differential methylation. In this section, we focus on the bioinformatics analyses of bisulfite-converted data, in particular WGBS and RRBS (see Fig. 3 for a general bioinformatics pipeline).

### Aligning bisulfite-converted reads and data visualization

The bisulfite sequencing data are generally processed with several steps, including adapter trimming [92], a quality assessment of reads [93–95], aligning reads to the reference genome [96–101] and methylation calling [102]. In particular, mapping bisulfite-converted reads is challenging due to reduced sequence complexity, asymmetric C to T alignments, and the fact that the bisulfite-converted Watson and Crick strands are not complementary to each other because bisulfite conversion occurs only at Cs (not G’s) [96, 103]. To address these issues, a number of alignment and post-alignment analysis tools have been developed (see Table 2 for a list of alignment tools and software for post-alignment analysis). Bisulfite sequencing aligners are mostly based on one of two algorithms: wild cards and three-letter algorithms. Wild card aligners substitute Cs with Ys in the reference genome, and reads with both Cs and Ts can then be aligned. This method results in higher genomic coverage together with a bias toward higher methylation levels [91]. However, the three-letter aligners convert all Cs in the reference genome and the read into Ts, and thus, standard aligners with lower mappability can be adopted due to reduced sequence complexity. For example, in the analysis of black truffle methylome, the bisulfite-treated reads were mapped 15–25 % less, comparing to the untreated [21]. In Table 2, we list 7 major bisulfite sequencing aligners along with their features. For example, BS Seeker 2 is a three-letter aligner that supports the local alignment and computational removal of potentially unconverted reads. The alignment profile can be visualized with tools such as the UCSC genome browser [104], WBSA [105], IGV [106] and Methylation plotter [107], which results in greater clarity at a single-base resolution across the genome.

**Table 2:** Bioinformatics tools for bisulfite sequencing data analysis

Function(s)	Software	Features	References
Quality trim	Cutadapt	Removes adapter sequences	[92]
Bisulfite sequencing aligner	Bismark	Three-letter aligner; supporting both Bowtie and Bowtie2	[96]
	BRAT-BW	Three-letter aligner for mapping and methylation calling	[97]
	BS Seeker 2	Three-letter aligner; supporting local alignment, and computational removal of unconversion reads	[98]
	MethylCoder	Three-letter aligner to be used with Bowtie or GSNAP	[99]
	GSNAP	Wild card aligner	[100]
	LAST	Wild card aligner wrapped in a general-purpose alignment tool	[101]
Data visualization	UCSC Genome Browser	Web-based genome browser allowing visualizing DNA methylation data (https://genome.ucsc.edu)	[104]
	WBSA	Web service for comprehensive analysis of WGBS and RRBS data and DMR finding (http://wbsa.big.ac.cn/)	[105]
	Integrative Genome Viewer (IGV)	Graphical genome browser to run locally on the user’s computer	[106]
	Methylation plotter	Web-based tool that plot up to 100 samples in lollipop or grid style (http://gattaca.imppc.org:3838/methylation_plotter)	[107]
Post-alignment data analysis	BSPAT	Summarizing and visualizing DNA methylation co-occurrence patterns Detecting allele-specific methylation Performing integrative analysis with genomic features such as histone modification	[108]
	GBSA	Sequencing quality assessment Methylation level scoring Data management and visualization	[93]
	MethGo	Calculating and plotting global methylation level Genome-wide methylation plot Calculating methylation level in different genomic regions Extracting SNP and CNV information from BS-seq data Profiling methylation at transcriptional factor binding sites	[109]
	SAAP-RRBS	Read quality assessment Alignment and methylation calling CpG annotation and reporting for high coverage and quality CpGs that could be visualized with IGV	[94]
	SMAP	Read quality assessment Alignment and methylation calling Differentially methylated cytosine detection with Chi-square test and DMR calling by Fisher’s exact test Detecting SNPs and allele-specific methylation	[95]
Identifying DMR	BSmooth	A pipeline includes alignment, quality control and data analysis; the DMR finding function adapts bump hunting on smoothed t-like score; supporting multiple testing correction	[110]
	methylKit	R package for clustering, sample quality visualization and DMR finding with logistic regression; supporting multiple testing correction	[112]
	methylSig	R package for DMR finding with likelihood ratio test; supporting multiple testing correction	[113]
	methylPipe	R package for DMR finding with Wilcoxon or Kruskal–Wallis paired nonparametric test; supporting multiple testing correction	[114]
	BiSeq	R package for DMR finding with Wald test; performing comprehensive RRBS data analysis; supporting multiple testing correction	[115]

*BRAT*-*BW* Bisulfite-treated Reads Analysis Tool (Burrows–Wheeler transform), *UCSC genome browser* University of California Santa Cruz Genome Browser, *WBSA* web service for bisulfite sequencing data analysis, *BSPAT* bisulfite sequencing pattern analysis tool, *GBSA* genome bisulfite sequencing analyser, *IGV* integrative genomics viewer, *SAAP*-*RRBS* streamlined analysis and annotation pipeline for RRBS data, *SMAP* streamlined methylation analysis pipeline

### Post-alignment data analysis

Bisulfite aligners will output aligned reads along with the methylation calling information of each C with sequence context information, e.g., the CGmap file in BS Seeker 2 [98]. Users can filter out sites with coverage, calculate the average methylation level and generate informative plots. Table 2 lists 5 post-alignment analysis tools, and each of these tools has specific functions, e.g., BSPAT can detect allele-specific methylation [108], SAAP-RRBS can extract the annotation of each C [94] and MethGo can convert context methylation levels into average and genome-wide plots, as well as extract SNP and CNV profiles [109].

### Detection of differentially methylated loci and regions

WGBS and RRBS generate methylation calls at each C as an estimate of the percentage of cells with methylation. Statistical tests are employed to identify differentially methylated loci in comparisons. For studies without replicates, Fisher’s exact test is generally adopted. A comparison with no replicates completely ignores within-group variations, resulting in an overstatement of the differences and a high false-positive rate. Hansen et al. described the need for biological replicates and developed BSmooth to effectively use low-coverage data with biological replicates and to determine DMRs [110].

DMRs are genomic regions that exhibit a different methylation status between two groups of samples. For example, Choufani et al. assessed genome-wide DNA methylation maps in human uniparental samples, a mature cystic ovarian teratoma (MCT) carrying the maternal genome and an androgenetic complete hydatidiform mole (AnCHM) carrying the paternal genome, as references to identify imprinted genes and DMRs. The comparison between the MCT and AnCHM successfully identified AXL as a new imprinted gene [111].

The identification of DMRs relies on both computational power for genome-wide screening and statistical testing. In Table 2, we included tools for implementing statistical methods in DMR screening [110, 112–115]. Generally, the DMR detection algorithm adopts a sliding window across the genome to survey candidate DMRs, and the most common approach is to perform Fisher’s exact test CpG-wise. To detect DMR, as the coverage of each sample may be different, only sites covered by all samples are comparable. To enable the comparison, the comparing statistics such as methylation difference, T-score from *T* test or *P* value is needed in the testing. In the BSmooth software, a beta-binomial is assumed to be the suitable model for replicated bisulfite sequencing data. The observation is assumed to be binomially distributed, whereas the methylated proportion at a particular site can vary across samples. The differences at an individual site could be small but may expand and persist across a region, which is a candidate DMR. Therefore, DMRs are determined with greater statistical power and are more informative. When comparing methylomes with weak differences, extending the testing scale from one C to a cluster of neighboring Cs can reduce the number of hypothesis tests to improve the statistical power [91] (e.g., BiSeq takes spatial correlation into account in DMR prediction [115]). Weak DNA methylation differences can be better measured by estimating the standard deviation from biological replicates to obtain more robust *P* values [91].

### Multiple testing in DMR detection

In addition, multiple testing is increased when many sites are simultaneously tested. In Table 2, we include a list of software that enables a correction for multiple testing.

Schmitz et al. performed a large-scale WGBS analysis in which DMRs from many Arabidopsis methylomes were detected [116]. They used the R package methylPipe to scan the genome with 100-bp windows [114], and the methylation level of the sites within a window was compared across all samples using a Kruskal–Wallis test. The *P* values were then adjusted for multiple testing using the Benjamini–Hochburg method, and only DMRs with an adjusted *P* value less than 0.01 were selected. In addition to the adjusted *P* value, a second criterion is used to ensure the differences, and the DMR has to exhibit an eightfold methylation difference between the two groups.

Gkountela et al. devised an in-house method to identify DMRs between each developmental stage during human PGC development [83]. In their comparisons among the ICM (inner cell mass), PGCs and AGCs (advanced germ cells), these authors identified candidate DMRs with at least an 80 % methylation difference in 200-bp windows. To evaluate the FDR, they generated simulated methylomes with the same read coverage per site as the real samples and reproduced the methylation level per site. The methylation levels were generated from a binomial model in which the parameters were the same for all the samples, i.e., all the simulated methylomes were equally methylated in both comparison groups. Any DMRs identified from the simulated methylomes were considered false positives. In total, Gkountela et al. found 3445 DMRs between PGCs and AGCs with an FDR < 0.001 %.

Robinson et al. reviewed some of the major DMR tools and discussed how the statistical significance was assessed [117]; users were advised to select the tool that satisfies their experimental design and data format. For example, most of the tools have been developed based on human and mouse studies; therefore, users studying other organisms should take the flexibility of the tools into account. The accommodation of different data types should also be considered, e.g., BiSeq supports RRBS only, whereas methylPipe supports RRBS and WGBS, as well as low-resolution DNA methylation data.

## Conclusions

This review provides an overview of the current techniques for the assessment of genome-wide DNA methylation and the identification of DMRs. The commonly used techniques are primarily based on restriction enzyme digestion, affinity enrichment and bisulfite treatment, coupled with either microarray or sequencing technologies. Because each technique has its own advantages and disadvantages, we summarize in Table 1 a comprehensive evaluation of each technique. In Fig. 2, we provide an overview of these experimental pipelines and their required DNA input amounts. The selection of a technique strongly depends on the research questions, cost, amount of input DNA and the expected degree of methylation changes [118]. In Table 1, readers can also learn from the biological examples in which the profiling techniques were used to determine the experiments that best fit their research topic. For example, for mammalian studies with large-scale samples, one should consider a targeted approach, such as MeDIP or RRBS rather than WGBS, which would allow multiple sample comparisons with limited cost and provide sufficient information from CpG-rich regions. If the study aims to investigate the first methylome of an organism, then WGBS with deep sequencing would be a more suitable method to obtain detailed information in coding regions and intergenic regions. The input DNA amount should also be considered when rare cell types or tissues are studied. To reveal the methylation state of undifferentiated stem cells without heterogeneity, single-cell approaches would be the best choice. The sequencing depth is a key parameter in DMR discovery; the greater the depth, the more power to discover DMRs. However, for studies with a large sample size such as disease-centered research studies, the distribution of limited resources should be considered, e.g., sequencing a few samples deeply or more samples less deeply. A balance may be reached by considering the profiling technique coupling with the data analysis that would provide precise and accurate DMR prediction with low coverage requirements.

The discovery of various forms of C modifications, namely 5hmC, 5fC and 5caC, further expand the efforts to map and quantify these low-abundance bases in different cell and tissue types [76]. The emerging SMRT and nanopore sequencing technologies have enabled the direct reading of C modifications without the pre-treatment of DNA and amplification; however, the throughput and accuracy must be substantially improved before these techniques become contenders against second-generation sequencing technologies [14].

In addition to providing underlying biological insights, DNA methylation assays have great potential for application to different fields, particularly medicine and forensic sciences [119, 120]. In medicine, these methodologies aid in the identification of epigenetic-based biomarkers for cancer and other epigenetic-related diseases, which serve as measurable indicators of biological conditions for predicting the presence or severity of a disease state or treatment response and further contribute to the development of clinical treatments and personalized medicine throughout life [120–124]. DNA methylation has been applied to the discrimination of fetal and maternal DNA in circulating cell-free DNA to obtain more pure fetal DNA for downstream analyses, such as chromosomal abnormality [125]. With improvements in low-input bisulfite sequencing and single-cell techniques, methylomes at an early embryonic developmental stage during pregnancy could be obtained to identify an abnormal fetus. Regarding applications in forensic sciences, DNA methylation analysis may be useful in the verification of DNA samples, body fluid identification and the estimation of ages and phenotypic characteristics [119]. The ongoing advancements in technology allow the development of more accurate and affordable methods for methylation analysis, such as with the application of single-cell noninvasive prenatal tests, and further enhance our understanding of the roles of DNA methylation and its underlying mechanism in disease progression and the modulation of DNA methylation in response to different environmental cues in different cell and tissues types.

## Abbreviations

5caC: 5-carboxylcytosine
5fC: 5-formylcytosine
5mC: 5-methylcytosine
5hmC: 5-hydroxymethylcytosine
6mA: N^6^-methyladenine
CGI: CpG island
DMR: differentially methylated region
ESC: embryonic stem cell
FDR: false discovery rate
hESC: human embryonic stem cell
hMeDIP: hydroxymethylated DNA immunoprecipitation
MethylCap-seq: methylated DNA capture by affinity purification sequencing
MRE: methylation-sensitive restriction enzyme
MBD: methyl-CpG-binding domain
MeDIP: methylated DNA immunoprecipitation
OxBS-seq: oxidative bisulfite sequencing
PGC: primordial germ cell
RRBS: reduced-representation bisulfite sequencing
SMRT: single-molecule real-time sequencing
TAB-seq: TET-assisted bisulfite sequencing
TE: transposable element
TET: ten-eleven translocation family
TSS: transcription start site
WGBS: whole-genome bisulfite sequencing
